# Human CARD9: A Critical Molecule of Fungal Immune Surveillance

**DOI:** 10.3389/fimmu.2018.01836

**Published:** 2018-08-06

**Authors:** Rebecca A. Drummond, Luis M. Franco, Michail S. Lionakis

**Affiliations:** ^1^Fungal Pathogenesis Section, Laboratory of Clinical Immunology and Microbiology (LCIM), National Institute of Allergy and Infectious Diseases (NIAID), National Institutes of Health (NIH), Bethesda, MD, United States; ^2^Laboratory of Immune System Biology (LISB), National Institute of Allergy and Infectious Diseases (NIAID), National Institutes of Health (NIH), Bethesda, MD, United States

**Keywords:** CARD9, fungi, primary immunodeficiency, C-type lectin receptors, candidiasis, neutrophils

## Abstract

CARD9 is a signaling adaptor protein that is involved in the transduction of signals from a variety of innate pattern recognition receptors, including the C-type lectin receptors and intracellular NOD receptors and nucleic acid sensors. As a result, CARD9 has been shown in animal models to be an important regulator of immunity to bacteria, fungi, and viruses. Studies in humans with autosomal recessive CARD9 deficiency have indicated a highly specific role for this molecule in the activation of antifungal immune responses in the central nervous system, the oral mucosa, and the skin. Moreover, CARD9-dependent functions have recently been indicated to modulate the development of autoimmunity, inflammatory bowel diseases, and cancer. In this mini-review, we highlight the recent studies that have identified several novel functions of CARD9 in various disease contexts, and we summarize the contemporary understanding of the genetics and immunology of human CARD9 deficiency.

## Introduction

Innate recognition of microbes by pattern recognition receptors (PRRs) is a critical first step in the defense against infection. To activate antimicrobial immunity, PRRs must initiate intracellular signaling cascades which control cellular responses, such as cytokine production, phagocytosis, and assembly of microbial killing complexes. Many PRRs use signaling molecules and adaptor proteins that are shared with other members of the same PRR family. As a result, deficiency of a shared signaling or adaptor protein often results in immune dysfunctions that are more severe than a deficiency in a single receptor and can have profound consequences for the control of infections (1).

Pathogenic fungi are predominantly recognized by the C-type lectin receptor (CLR) and toll-like receptor (TLR) families, of which many members couple to the signaling adaptor proteins CARD9 and MyD88, respectively, in order to activate their functions and initiate defense against fungal invasion (2). Although both CLRs and TLRs bind to and activate antifungal immune responses, the phenotypic consequences of human deficiencies in either of the two shared signaling adaptors have demonstrated that CARD9 plays a specific, superior role in the control of fungal diseases in humans compared to that of MyD88. Indeed, patients with genetic deficiency in CARD9 exhibit a primary immunodeficiency disorder (PID) which manifests as an extreme susceptibility to fungal infections, but not bacterial, viral, or parasitic infections, and is the only PID described to date that specifically predisposes to fungal diseases without other infectious or non-infectious sequelae (3). In contrast, MyD88 deficiency results in the development of life-threatening pyogenic bacterial infections without the spontaneous development of fungal disease (4). The highly specific susceptibility to fungi in CARD9-deficient patients further extends to the fungal species and organs affected, indicating that CARD9 is required for organ-specific antifungal immune responses of which we are only now beginning to define. Furthermore, animal models and genome-wide analyses of human single-nucleotide polymorphisms (SNPs) have indicated that CARD9 may also function to promote immunity to other pathogens and contribute toward autoimmune and hyperinflammatory disorders. Thus, CARD9 is a multi-functional signaling protein involved in many aspects of the immune system.

In this review, we discuss recent research that has unveiled the many possible functional roles of CARD9, predominantly defined using animal models. Finally, we discuss the genetics of human CARD9 deficiency and highlight the critical role for this molecule in antifungal immune surveillance in humans.

## The Multi-Functional Roles of CARD9

### CARD9 Is a Critical Activator of Antifungal Immune Responses

**Figure 1:**
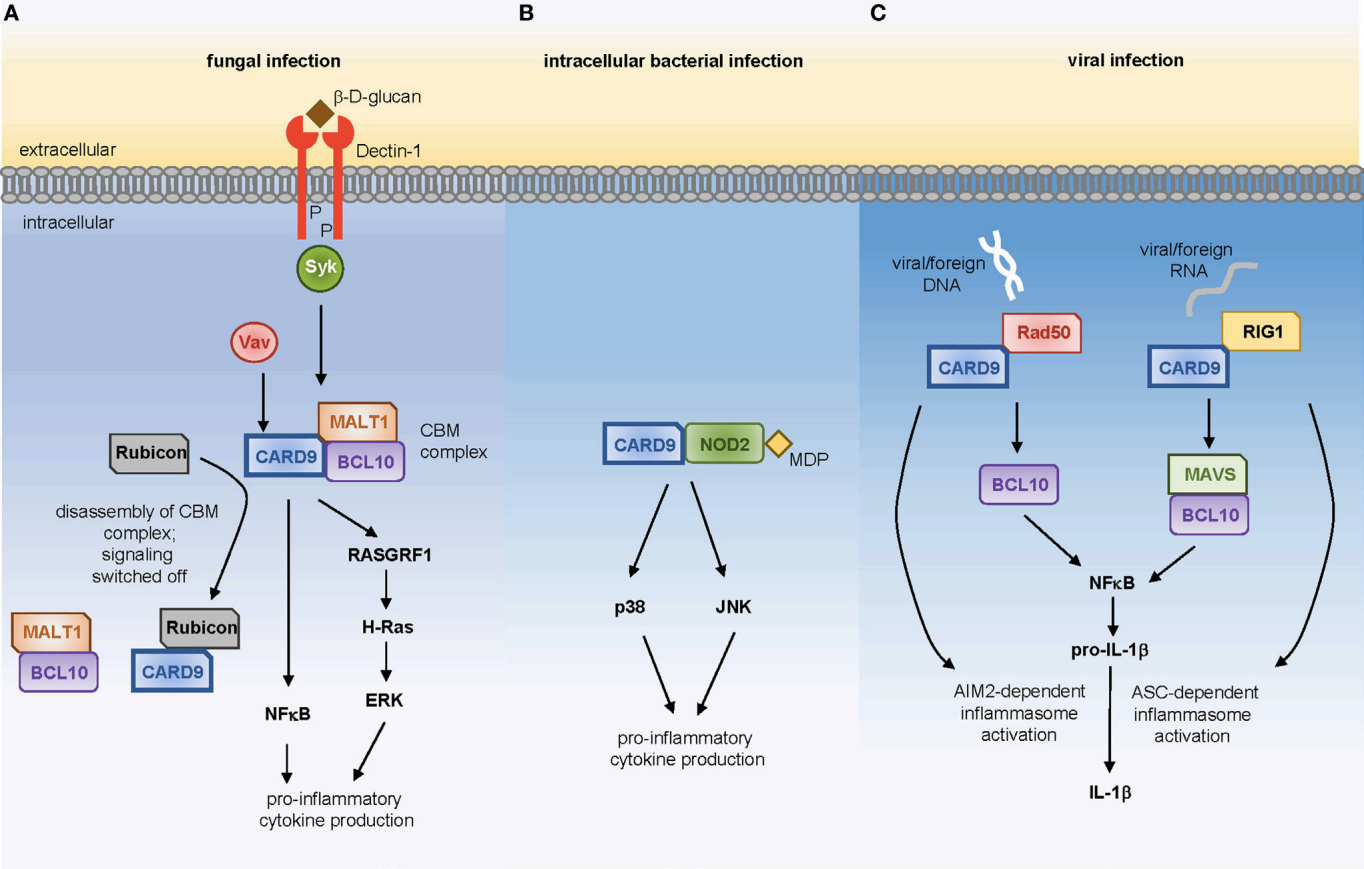
CARD9-dependent signaling in response to **(A)** fungal ligands *via* Dectin-1, **(B)** muramyl-dipeptide derived from intracellular bacterial pathogens, or **(C)** viral DNA or RNA.

Many of the prototypical PRRs that recognize common components of fungal cell walls are members of the CLR family, and include Dectin-1, Dectin-2, Dectin-3, Mincle, and the Mannose Receptor (CD206). Many of these receptors initiate intracellular signaling cascades that are CARD9-dependent, the best studied of which is the Syk-dependent pathway downstream of Dectin-1 (Figure 1A). In brief, ligation of Dectin-1 by β-glucan (the fungal ligand for this receptor) results in recruitment of Syk kinase and the formation of the CBM signalosome, composed of CARD9, BCL10, and MALT1 (5). Activation of CARD9 function requires Vav proteins, of which there are three isoforms (Vav1, Vav2, and Vav3). Deletion of Vav1-3 in mice results in a phenocopy of CARD9-deficiency, indicating that Vav-mediated activation of CARD9 is a critical step in the induction of protective antifungal immunity. In line with this, human *VAV3* SNPs are enriched in patient cohorts with candidemia (6). Activated CARD9 then leads to the production of inflammatory mediators, such as IL-6, IL-12, GM-CSF, TNF, and IL-1β *via* the activation of NFκB and ERK, the latter of which occurs *via* a RASGRF1–H-Ras pathway (7). These CARD9-dependent signaling pathways are regulated by Rubicon, a protein best known for its functions in autophagy (8). Rubicon competitively binds to CARD9 which results in the disassembly of the CBM complex, thus switching off signaling and preventing excessive inflammatory responses. However, modulation of Rubicon expression levels using lentiviral vectors in mice demonstrated that a reduction in Rubicon expression could help promote fungal clearance and survival by enhancing CARD9-dependent antifungal immune responses, at least in acute infection models (8). Compared to other PRR families, the molecular signaling events that occur upon CLR ligation are poorly defined. Yet, understanding these pathways will be critical to determine how iatrogenic interventions that manipulate these pathways affect vulnerable patients. For example, the recently introduced Syk inhibitors for hematological malignancies and graft-versus-host disease (9, 10), may predispose to the development of dangerous invasive fungal infections in patient cohorts already at risk for these diseases.

Animals with a genetic deletion of Card9 are susceptible to challenge with a variety of fungal species, including *Candida albicans, Aspergillus fumigatus, Cryptococcus neoformans*, and some rarer dematiaceous fungi (11–14). In each of these models, *Card9^−/−^* mice generally exhibit reduced inflammatory cytokine production, which contributes to the inability to control fungal growth within infected organs. Protective antifungal cytokines that appear critically dependent on CARD9 function include IL-6, TNF, and IL-1β. In humans, CARD9 deficiency predisposes to a smaller range of pathogenic fungi (discussed below) indicating that CARD9-dependent functions in humans may be more context-dependent than in the mouse.

Despite the central position of CARD9 in the CLR signaling pathway, ablation of CARD9 function does not result in complete abrogation of antifungal immune responses (in either mice or human), indicating that CARD9-dependent mechanisms are required only for certain protective processes that may be specific to the cell type in question. Indeed, the dependency on CARD9 for NFκB activation has been shown to vary among different types of myeloid cells (7), including neutrophils, macrophages, and dendritic cells (DCs) (13). Neutrophils are the most important effector cell in the defense against systemic *C. albicans* infections, since neutropenia is a significant independent risk factor for the development of and suffering worse outcome from these infections in humans, and neutrophil depletion significantly reduces survival to *C. albicans* challenge in mice (2, 15). Many intrinsic neutrophil functions, including phagocytosis, production of reactive oxygen species (ROS), and chemotaxis appear largely independent of CARD9 for a variety of fungal species, shown with *ex vivo* studies using human neutrophils (16–19). In contrast, for ROS-independent fungal killing, CARD9 is required for the killing of unopsonized yeast cells as part of a PI3Kγ-dependent pathway, whereas killing of opsonized *C. albicans* yeast largely requires FcRγ and PKC and is independent of CARD9 (20). These two distinct fungal killing pathways utilized by neutrophils helps to explain the observation that neutrophils isolated from CARD9-deficient patients exhibit a selective killing defect toward unopsonized yeast (18, 19). This defect may partly contribute toward the fungal central nervous system (CNS)-tropism observed in CARD9-deficient patients (see below), since opsonization is naturally low in the CNS and thus CARD9-dependent killing of unopsonized cells would be particularly critical at this site. However, human neutrophil killing against the invasive filamentous *Candida* forms observed in tissue appears intact, whether opsonized or unopsonized (19). In addition, we and others have shown that a critical function of CARD9 in antifungal defense is promoting neutrophil recruitment specifically into fungal-infected organs *via* CXC chemokine production in both mice and humans (19, 21), which appears to largely contribute toward the organ-specific manifestation of fungal diseases observed in CARD9-deficient humans.

In experimental *C. albicans* CNS infections in mice, CARD9 promotes neutrophil recruitment in a fungal- and brain-specific manner (19). In this situation, CARD9 is required for the appropriate induction of CXC chemokines in the CNS by resident macrophages (i.e., microglia) and glia cells, as well as recruited neutrophils. Therefore, CARD9 controls local inflammatory chemokine production in addition to a neutrophil-intrinsic positive feedback chemotaxis loop in the brain (19). CARD9-dependent production of neutrophil-attracting chemokines is also evident during pulmonary infection with *A. fumigatus* in mice (14), and we recently reported CARD9-deficient patients who developed extrapulmonary *A. fumigatus* infection that was associated with a lack of neutrophil accumulation at the infected site (21). Moreover, reduced production of CXC chemokines resulting in a lack of neutrophil recruitment has been described in chronically fungal-infected subcutaneous tissue in experimental phaeohyphomycosis models (22). Therefore, CARD9 appears to be a central regulator of neutrophil recruitment to specific organs during invasive fungal infection.

### CARD9 and Anti-Bacterial Immunity

Stimulation of *Card9^−/−^* myeloid cells with purified bacterial products has revealed that CARD9 is required for inflammatory cytokine production in response to specific bacterial stimuli. IL-6 production following stimulation with peptidoglycan or muramyl-dipeptide (MDP), predominant components of Gram-positive bacterial cell walls, is highly dependent on CARD9 whereas responses to LPS are CARD9-independent (23). Recognition of MDP is largely controlled by the intracellular NOD2 receptor, to which CARD9 couples in order to drive activation of p38 and JNK kinases (Figure 1B), thus promoting immunity to intracellular bacterial pathogens including *Listeria monocytogenes* (23) and *Mycobacterium tuberculosis* (24).

In addition to activating innate immune responses to intracellular bacteria, other studies have indicated that CARD9 may be involved in the induction of adaptive immunity to these pathogens. Mouse T-cells specific for flagellin were shown to require Syk-CARD9 signaling for their activation, which was mechanistically linked to TLR5-dependent antigen presentation by CARD9-expressing DCs (25). Polarization to the Th17 lineage also depends on CARD9 in the context of gastrointestinal (GI) bacterial infection (26), similar to what has been described for the generation of Th17 responses in the fungal-infected oral mucosa (27). Moreover, humoral immunity mediated by B-cells has also been shown to be affected by deletion of Card9, since bacterial-specific IgG production during GI infection is significantly reduced in *Card9^−/−^* animals (28). Thus, animal models have demonstrated that Card9 can play distinct protective roles in immunity to intracellular bacterial pathogens, however, the relevance of these functions in humans remains to be determined given that CARD9-deficient patients do not appear susceptible to bacterial infections.

### CARD9 and Antiviral Immunity

Studies delineating the molecular pathways controlling IL-1β activation and secretion by myeloid cells in response to viral infection have identified CARD9 as a key regulator of these responses (Figure 1C). Following viral infection or transfection with foreign DNA, CARD9 directly interacts with Rad50, a cytosolic DNA sensor, and these CARD9-Rad50 complexes subsequently recruit BCL10 to promote IL-1β secretion. Using a vaccinia virus infection model, *Card9^−/−^* animals were shown to produce reduced levels of IL-1β following infection which negatively impacted the ability of these animals to generate an appropriate antiviral CD8^+^ T-cell response (29). CARD9 is also required for immunity to retroviruses by transducing RIG-1-dependent signals *via* MAVS and BCL10 to activate NFκB and transcription of pro-IL-1β, which in turn is processed into active IL-1β by ASC-dependent activation of the inflammasome (30). As shown for antifungal immunity, Rubicon can also regulate these CARD9-dependent antiviral functions, since enhancing Rubicon expression levels was found to significantly skew host immunity in favor of pathogen resistance (8).

### CARD9 and Autoimmunity

Recent studies have demonstrated potential novel roles of CARD9 in autoimmune diseases using animal models, providing interesting new insights into how this protein functions in organ-specific inflammation. During experimental uveitis, Mincle-mediated activation of CARD9 is required for the recruitment of pathogenic Th1 and Th17 cells (31), and it was later shown that fungal antigens could exacerbate the development of this disease and this occurred *via* Dectin-2-CARD9 signaling (32). In line with the critical role for CARD9 in neutrophil recruitment, *Card9^−/−^* animals showed reduced production of neutrophil-targeted chemokines, such as CXCL1, CXCL2, and CCL3, resulting in decreased accumulation of neutrophils to the arthritic joint (33). As a result, *Card9^−/−^* animals are protected from autoantibody-induced arthritis, and this protection was mapped to neutrophils using neutrophil-specific Card9-deficient mice (33). Population-based human studies have indicated that *CARD9* genetic variation may modulate the risk of development of inflammatory bowel diseases (IBD; discussed below), ankylosing spondylitis (34), IgA nephropathy (35), and primary sclerosing cholangitis (36), and more studies are required to determine the role of the CLR/CARD9 axis in organ-specific autoimmune disease development.

### CARD9 and Cancer

CARD9-dependent signaling in tumor development and metastasis has been revealed in several recent studies using animal models and cell lines. Patients with IBD are at risk for the development of colonic cancers. Human SNPs in *CARD9* have been identified as risk factors for IBD by several GWAS-based studies (discussed below), and animal models have shown that CARD9 promotes the production of pro-inflammatory cytokines IL-1β and IL-22 in the gut during active colitis, which in turn drives colonic tumor growth (37). Other work has additionally shown that metastasis of colonic tumor cells to the liver depends on CARD9 signaling. CARD9 is highly expressed by tumor-infiltrating macrophages and has been shown to be a critical modulator of the polarization of these cells toward a highly inflammatory metastatic-inducing phenotype (38).

In addition to colonic cancer, CARD9 signaling has also been implicated in the inappropriate activation of renal cell carcinoma (RCC) cells. Mutation affecting the tumor suppressor gene *VHL* (most often somatic, but occasionally involving germline cells) is a major hallmark of RCC, which in turn causes inappropriate activation of NFκB and c-Jun. CARD9 is a mechanistic link between VHL inactivation and the activation of these inflammatory transcription factors (39, 40). Direct interaction between CARD9 and VHL is required for C-terminal phosphorylation of CARD9 by the kinase CK2, which limits CARD9 activity and NFκB activation. Reducing CARD9 expression with a silencing RNA approach in *VHL^−/−^* cells lowered NFκB activity to wild-type levels, and significantly reduced the tumorigenic potential of these cells (40). Similarly, the loss of VHL promotes an additional pro-inflammatory CARD9-dependent pathway that results in the activation of JNK signaling and c-Jun activity (39). Collectively, these studies provide the intriguing possibility that inappropriate CARD9 activation contributes to the development of certain cancers and may be a novel target of future therapies for these diseases.

## The Genetics and Clinical Spectrum of Human *CARD9* Mutations

Despite the wide range of CARD9-dependent functions identified in animal models and the potential influence of this protein in several disease scenarios, loss-of-function mutations in human *CARD9* have unequivocally demonstrated the importance of CARD9 in antifungal immunity. Human CARD9 deficiency is characterized by the spontaneous development of fungal infections that predominantly localize to the oral mucosa, CNS, bone, and subcutaneous tissues, and often involves specific families of pathogenic fungi including *Candida* species (CNS, bone, and mucosal disease) and dark-walled molds and yeast-like fungi (e.g., *Aspergillus, Exophiala*, and *Phialophora*) that localize to the CNS, skin, bone, and abdominal organs (3, 21, 41–43). The underlying mechanisms that cause susceptibility to fungal diseases in CARD9-deficient patients is not well understood, and could be related to the poor production of inflammatory cytokines and chemokines in response to fungal agonists (13, 18, 19). Indeed, a cohort of French-Canadian CARD9-deficient patients were successfully treated with recombinant GM-CSF therapy, which the authors show corrected defective GM-CSF responses and ERK signaling in CARD9-deficient myeloid cells (43), while another study used G-CSF therapy to correct defective IL-17 responses in a CARD9-deficient patient (44). These studies indicate that replacing cytokines that are classically associated with antifungal defense may be an appropriate therapy option for human CARD9 deficiency. However, we recently showed that these approaches may not be effective for all patients which are potentially related to the variety of *CARD9* mutations observed (see below). Our data indicated that although different *CARD9* mutations disrupt GM-CSF production by myeloid cells, the impact of these mutations on ERK activation varied with mutation and appeared to correlate with the efficacy of GM-CSF therapy in these patients (45). Thus, treatment options for human CARD9-deficiency are still limited, and further investigation into the mechanisms that cause fungal susceptibility in these patients is warranted. In addition, the organ- and fungal species-specific nature of CARD9-deficiency disease is also poorly understood. As discussed above, we and others have shown that defects in neutrophil recruitment are likely the major contributing factor toward the organ-specific nature of the disease, however, other contributing roles of tissue-resident macrophages, stromal cells, and/or additional functional deficits in recruited inflammatory cells remain to be fully explored.

Although many CARD9-deficient patients have similar clinical presentations, there is diversity in the genetic mutations underlying the condition [see Ref. (3) for a table of all reported human *CARD9* mutations]. More than 15 missense and nonsense mutations in *CARD9* have now been described in the coiled-coil and CARD domains, as well as the promoter region (3). Inactivation of both alleles appears to be necessary for the occurrence of disease, so the condition follows an autosomal recessive mode of inheritance. *De novo* variants in *CARD9* have also been reported in patients with debilitating fungal infections of the eyes, bone, and skin (46, 47). Interestingly, although some of the reported *CARD9* mutations have been identified in unrelated patients, there are instances where the same mutation does not give rise to a similar clinical phenotype (3). For example, homozygosity for the Q298X mutation has been reported to give rise to *Candida* meningoencephalitis and deep dermatophytosis in different patients (41, 48), while patients with very similar clinical presentations can have mutations in different parts of the *CARD9* gene (47). Therefore, it is currently unclear which mutations predispose to which clinical phenotypes and whether there is any overlap, and this will require more clinical descriptions of CARD9-deficient patients and further investigations into CARD9-dependent immune signaling and functions, especially in humans.

Like many other genes that are associated with Mendelian traits, human *CARD9* is intolerant to sequence variation. To quantify gene-level intolerance to functional genetic variation, Petrovski et al. proposed a Residual Variation Intolerance Score (49). Of the nine CARD genes for which a score was calculated in that study, only three (*CARD9, CARD10*, and *CARD11*) had negative scores, suggesting low tolerance to functional genetic variation. In fact, *CARD9* had lower tolerance to variation than 82% of the 16,956 genes for which a score was calculated in the study.

In addition to deleterious mutations which predispose to fungal infection, there have been reports of genetic variants of *CARD9* in humans that associate with autoimmune disease. The CARD9 single-nucleotide polymorphism S12N (rs4077515) was recently shown to be highly enriched in patients with allergic bronchopulmonary aspergillosis, which the authors linked to RelB activation by this CARD9 variant that subsequently activated IL-5 production by alveolar macrophages and drove pathogenic eosinophil recruitment and Th2 responses within the lung (50). Another example is the CARD9 variant in which the C-terminal region is truncated (51, 52). One of these truncated variants of CARD9 (S12NΔ11 or *CARD9*Δ11) is strongly associated with protection against IBD, which is mechanistically linked to the inability of these variants to be activated by TRIM62, a ubiquitin ligase that is required for CARD9 activation and subsequent pro-inflammatory cytokine production. While the lack of TRIM62-mediated activation is protective in the context of inappropriate intestinal inflammation and may represent a target for therapeutic intervention in IBD (53), these mutations would be predicted to negatively affect antifungal immunity, since *Trim62^−/−^* animals are unable to control fungal growth and succumb to infection significantly faster compared to their wild-type counterparts (52). Intriguingly, in human cell lines, *CARD9*Δ11 variants act in a dominant-negative fashion (52), raising the possibility of the existence of humans who may be heterozygous for dominant-negative *CARD9* mutations in the C-terminal region and yet may present with functional CARD9-deficiency. This possibility should be considered by clinicians looking for the underlying cause of an extreme fungal infection in an otherwise immunocompetent patient.

While the *CARD9*Δ11 variant is protective in IBD, other lines of evidence suggest a role for *CARD9* in the pathogenesis of the disease. Specifically, there are other *CARD9* variants that are enriched in patient cohorts with IBD (51) indicating that dysregulated CARD9 function can have profound consequences for immune homeostasis in the gut. The *CARD9* human SNP, S12N, is associated with increased *CARD9* mRNA expression and IBD development (51, 54), but not fungemia (55), thus supporting the notion that CARD9-dependent functions in the immune system are context-dependent. However, many studies analyzing the functions of CARD9 in the context of GI inflammation have discovered links between the development of GI disorders and fungal commensals, indicating that CARD9-dependent functions in innate fungal defense may also be important in gut-related diseases. For example, CARD9 deficiency has been associated with an over-representation of fungi within the microbiota (56), and there have been reports of CARD9-deficient patients developing colitis caused by invasive intestinal infection with *Candida glabrata* (48), and most recently by the β-glucan-containing microalgae *Prototheca zopfii* (57). These studies suggest that, at least partially, CARD9 may function to control fungal microbes in the gut and prevent infection-related disease in these tissues, which might explain the strong association between human *CARD9* genetic variants and the development of IBD-related disorders.

## Concluding Remarks

The significance of CARD9 and its signaling partners in antifungal defense has been an important realization for the field in the past two decades. A comprehensive understanding of the CARD9-dependent signaling pathways and their relevance for disease processes will be required to (1) utilize CARD9 as a future therapeutic target and (2) prevent and treat possible side-effects of immune-based therapies for cancer and autoimmunity. In particular, the non-fungal-related functions of CARD9 warrant further study, especially to understand the relevance of these functions in humans who carry genetic variants of *CARD9*, which are currently only partially defined yet have the potential to significantly influence CARD9-dependent functions. The study of human CARD9 deficiency has yielded novel insights into how this adaptor signaling molecule functions to protect against invasive fungal diseases and, importantly, these findings point to potential new avenues for the development of immune-based treatments for invasive fungal infections, which represent a global clinical challenge.

## Author Contributions

All authors contributed in writing the mini-review.

## Conflict of Interest Statement

The authors declare that the research was conducted in the absence of any commercial or financial relationships that could be construed as a potential conflict of interest.
